# Quantitative analysis of gait and balance using deep learning on monocular videos and the timed up and go test in idiopathic normal-pressure hydrocephalus

**DOI:** 10.3389/fnagi.2025.1644543

**Published:** 2025-10-14

**Authors:** Hee-Jin Cho, Sangwook Kim, Hosang Yu, Sungmoon Jeong, Kyunghun Kang

**Affiliations:** ^1^Kyungpook National University Chilgok Hospital, Daegu, Republic of Korea; ^2^Research Center for Artificial Intelligence in Medicine, Kyungpook National University Hospital, Daegu, Republic of Korea; ^3^AICU Corp., Daegu, Republic of Korea; ^4^Department of Medical Informatics, School of Medicine, Kyungpook National University, Daegu, Republic of Korea; ^5^Department of Neurology, School of medicine, Kyungpook National University, Daegu, Republic of Korea

**Keywords:** idiopathic normal pressure hydrocephalus (INPH), timed up and go test (TUG), gait analysis, deep learning, video-based assessment, fall risk prediction

## Abstract

**Background:**

A vision-based gait analysis system using deep learning algorithms for simple monocular videos was validated to estimate temporo-spatial gait parameters in idiopathic normal-pressure hydrocephalus (INPH) patients. The Timed Up and Go (TUG) test has been used to reflect risk of falling in INPH patients. The aims of the study were (1) to investigate relationships between temporo-spatial gait parameters measured by a vision-based gait analysis system using monocular videos and TUG scores and (2) to determine whether an automated machine learning model based on these gait parameters could predict falling risk in INPH patients.

**Methods:**

Gait data from 59 patients were collected from the vision-based system. All patients were also evaluated with the TUG test. A TUG time of ≥13.5 s was used as a cut-off to identify potential fallers.

**Results:**

Timed Up and Go scores were negatively correlated with gait velocity, cadence, stride length, and swing phase. TUG scores were positively correlated with step width, stride time, stance phase, double-limb support phase, stride time variability, and stride length variability. The area under the curve for predicting falling risk using the automated machine learning-based model was 0.979. We found that velocity was the most important factor in predicting falling risk with the interpretable method called SHapley Additive exPlanations.

**Conclusion:**

This study identified important associations between gait parameters measured by vision-based gait analysis and TUG scores in INPH patients. An automated machine learning model based on gait parameters measured by vision-based gait analysis can predict falling risk with excellent performance in INPH patients. We suggest that our vision-based gait analysis method using monocular videos has the potential to bridge the gap between laboratory testing and clinical assessment of gait and balance in INPH patients.

## Introduction

Idiopathic normal-pressure hydrocephalus (INPH) is a treatable neurologic disorder characterized by ventricular dilatation (Evans’ index >0.3), normal cerebrospinal fluid (CSF) pressure at lumbar puncture, and a symptom triad of cognitive impairment, gait disturbance, and urinary dysfunction (Relkin et al., 2005). Gait and balance disturbances are core symptoms in patients with INPH and these disturbances lead to an increased risk of falling (Nikaido et al., 2019). And several studies have reported fall rates of 60%–80% in patients with INPH (Nikaido et al., 2018, 2019). One simple and commonly used measurement in gait function is the 10-meter walking test, in which the patient is asked to walk at their normal pace for 10 meters (Sundstrom et al., 2022). The output is simply the time needed to complete the task (Sundstrom et al., 2022). The Timed Up and Go (TUG) test incorporates components of gait and balance control, and has primarily been used to estimate risk of falling in elderly populations (Sundstrom et al., 2022). The TUG test is considered to be more comprehensive than the 10-meter walking test (Sundstrom et al., 2022). For the TUG test, patients are instructed to get up from an armchair, walk 3 meters at a safe and comfortable pace, turn around, walk back to the chair and sit down (Sundstrom et al., 2022).

To analyze patients’ gait, several marker-based measurement systems such as Vicon have been proposed (Chromy et al., 2025). These methods provide relatively precise gait measurements, but calibration steps are required before clinical use, and dedicated calibration devices as well as wide spaces are necessary. 3D motion capture systems based on inertial sensors such as gyroscopes or magnetometers have also been developed and used for gait analysis (Cloete and Scheffer, 2008). However, in addition to the calibration burden, wearable attachments to the human body can easily disturb natural movement and may alter gait patterns. Pressure-sensitive walkway systems such as GAITRite (Vítečková et al., 2020) enable precise analysis of gait patterns in the temporo-spatial domain. However, the restriction of the walking area on devices like force plates provides limited mobility, and these systems require expensive equipment and experienced operators. Video sensors provide a rich source of information that can be used for gait analysis (Wang et al., 2013; Jeong et al., 2021). Additionally, vision-based gait analysis systems offer remote and continuous monitoring with high accessibility due to their non-invasive characteristics and cost-effective setup. Recent evidence indicates that vision-based gait analysis using artificial intelligence algorithms can be used to validly assess stride dynamics during walking (Clark et al., 2013; Jeong et al., 2021). Further, in our recent study using the GAITRite gait analysis system as a reference system, a vision-based gait analysis method using monocular videos was proposed to properly estimate temporo-spatial gait parameters by leveraging deep learning algorithms in INPH patients (Jeong et al., 2021). Our vision-based gait analysis system demonstrated a strong correlation with the GAITRite gait analysis system across 11 gait parameters, providing comparable data for assessing gait dysfunction in INPH (Jeong et al., 2021). The vision-based gait analysis system can provide clinicians with a low-cost, non-intrusive, and easy-to-use system for quantitative gait analysis (Jeong et al., 2021). Our method, which uses a commodity camera, may improve accessibility to quantitative gait analysis in clinical settings and at home.

The aims of the study were (1) to investigate relationships between temporo-spatial gait parameters measured by a vision-based gait analysis system using monocular videos and TUG scores and (2) to determine whether an automated machine learning model based on these gait parameters could predict falling risk in INPH patients.

## Materials and methods

### Participants

Study participants were prospectively recruited from patients at the Adult Hydrocephalus Clinic of Kyungpook National University Chilgok Hospital, South Korea between October 2022 to September 2024. INPH diagnoses were made using criteria presented by Relkin et al. (2005). According to Relkin et al. (2005) criteria, which specify a minimum symptom duration of 3–6 months, the inclusion criteria for study participants were set as follows: 6 months progression or longer of gait disturbance along with either cognition or urinary symptoms, >40 years of age, and normal CSF opening pressure. Brain MRI showed ventricle expansion (Evans’ ratio >0.3) for all study participants, with no CSF flow obstruction. Exclusion criteria were as follows: patients with a hospitalization history of a significant psychiatric disorder, stroke, recent history of extensive alcohol use, or history of metabolic, neurological, or neoplastic dysfunctions that could show dementia symptoms. No participant in the study showed evidence of intracerebral hemorrhage, meningitis, head trauma, or another potential cause of hydrocephalus.

This study protocol was approved by the Institutional Review Board of Kyungpook National University Chilgok Hospital. All methods and procedures were performed in accordance with relevant guidelines and regulations. All study participants gave informed and written consent for the study, including information related to clinical data.

### Assessing illness severity

Comprehensive clinical scales for each INPH patient in the study was determined in the following manner. General cognition was evaluated with the Korean-Mini Mental State Examination (K-MMSE) (Kang et al., 1997). The Frontal Assessment Battery (FAB) was used to ascertain frontal lobe symptoms (Dubois et al., 2000). The total FAB score ranged from 0 to 18, with a higher score meaning better performance. Gait assessment included performance results on the TUG test and 10-meter walking test (Podsiadlo and Richardson, 1991; Rossier and Wade, 2001; Kubo et al., 2008). The TUG test measures the length of time it takes a patient sitting in a chair to stand up, walk forward 3 meters, and return to a seated position. The TUG test has been used to predict the risk of falling, and a TUG time of ≥13.5 s has been suggested as a cut-off to identify individuals at increased risk (Sundstrom et al., 2022). In this study, individuals with a TUG time of 13.5 s or more were considered to be at increased risk of falls (Sundstrom et al., 2022). While the cut-off of ≥13.5 s for the TUG test was originally proposed in community-dwelling elderly populations, this threshold has also been commonly used in studies including INPH patients (Yamada et al., 2019; Sundstrom et al., 2022). In our study, we therefore adopted this cut-off.

### Quantitative gait assessment

For gait assessment, we utilized a vision-based gait measurement system that we developed in our previous study (Jeong et al., 2021). All participants were told to walk barefoot at a reasonable and self-selected speed without the use of any walking aid. The process was repeated 4 times to obtain sufficient data for analysis. All patients were given time to rest between walking trials when requested to avoid fatigue. A researcher walked alongside each patient to ensure safety. The system’s camera was positioned 0.5 meters above the ground, either in front of or behind the participants, and recorded videos of patients walking a distance of 5 meters. By pipelining a YOLO v3 detector and ResNet18-based convolutional neural network (CNN) with PyTorch (He et al., 2016; Redmon and Farhadi, 2018; Paszke, 2019), the system detected and analyzed walking motion of the patient and yielded temporo-spatial gait measurements. Spatiotemporal gait parameters were determined using the system as follows: stride length, step width, gait velocity, cadence, toe in/out angle, stride time, stance phase (%), swing phase (%), double-limb support phase (%), coefficient of variation (CV) for stride time, and CV for stride length.

### Construction and evaluation of predictive models

Based on measured gait parameters, we applied automated machine learning (AutoML) using Mljar to predict fall risk (High or Low) given 11 gait parameters (Hutter et al., 2019; Płońska and Płoński, 2021). The AutoML procedure of Mljar consists of 2 stages: model search with cross-validation and ensemble learning. In the first stage of model search, the base models used were linear regression, decision tree, Random Forest, and XGBoost (Breiman, 2001; Chen and Guestrin, 2016; Breiman et al., 2017). Note that although deep learning models have achieved great success with unstructured signals like vision or sound, for structured tabular datasets, methods like Random Forest or XGBoost demonstrate superior results compared to deep learning models (Shwartz-Ziv and Armon, 2022). For the AutoML procedure, 5-fold cross-validation was applied with stratification strategy to consider class imbalance. After finding feasible models, the second stage of AutoML constructed an ensemble model to combine feasible models to build the high-performance model. The models selected for the ensemble were 3 Random Forests and a XGBoost, and our results were reported with the ensemble model. SHAP (SHapley Additive exPlanations) analysis is based on Shapley values from Game Theory, and calculates local contributions of each input feature of a sample to the corresponding model output (Lundberg et al., 2020). Since SHAP values for each sample are just local explanations for the sample, the importance of an input feature is generally defined by the absolute mean of SHAP values of the feature across all samples. To analyze the importance of gait parameters on fall risk prediction, we applied this same definition of importance.

## Results

Fifty-nine INPH patients constituted the final sample for analysis. Table 1 lists clinical and demographic parameters of INPH patients. The study participants included 38 men and 21 women; the mean age was 75.3 ± 5.5 years.

**TABLE 1:** Demographic data and clinical characteristics of INPH patients. Values denote number (%) or mean ± standard deviation.

Statistics	Total	High-risk for falling (*n* = 44)	Low-risk for falling (*n* = 15)
Gender, male	38 (64.4)	25 (56.8)	13 (86.7)
Age (year)	75.3 ± 5.5	76.6 ± 5.0	71.4 ± 5.5
K-MMSE	21.2 ± 5.6	20.2 ± 5.6	24.2 ± 4.2
FAB	10.3 ± 3.7	9.6 ± 3.6	12.3 ± 3.3
Timed up and go test	22.1 ± 20.4	25.5 ± 22.7	12.0 ± 1.3
10-meter walking test	20.7 ± 23.2	24.0 ± 26.1	11.1 ± 2.3

K-MMSE, Korean version of mini-mental state examination; FAB, frontal assessment battery.

### Correlations between clinical measures and gait parameters measured by the vision-based gait analysis system in INPH

**FIGURE 1:**
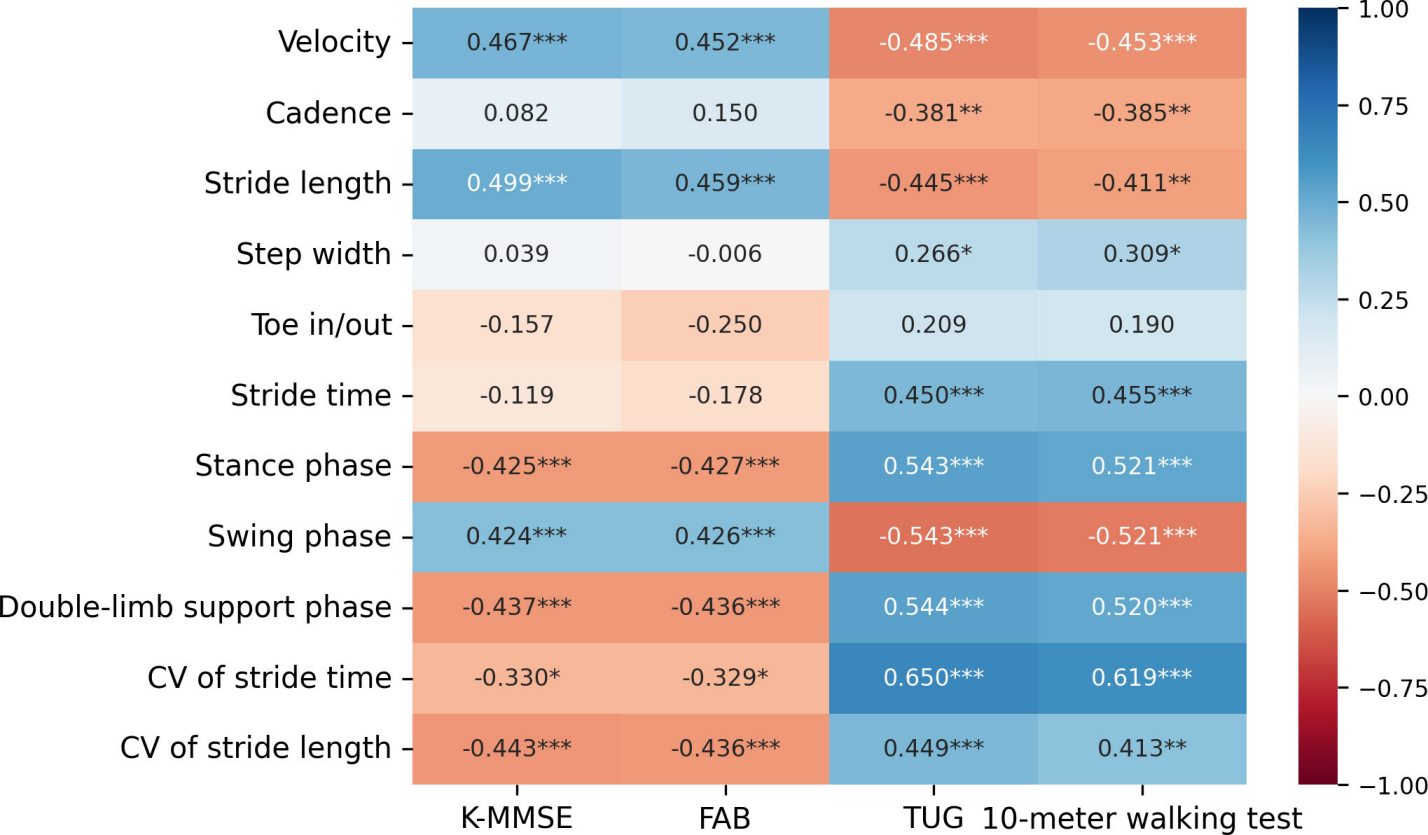
Pearson’s correlation coefficients between clinical measures and gait parameters measured by the vision-based gait analysis system in INPH. Heatmap shows Pearson’s correlation coefficients. The colorbar provides a visualization of the strength and direction of the correlation. ***P < 0.001, **P < 0.01, *P < 0.05. CV, coefficient of variability; K-MMSE, Korean version of mini-mental state examination; FAB, frontal assessment battery; TUG, timed up and go test.

The TUG and 10-meter walking test scores were negatively correlated with gait velocity, cadence, stride length, and swing phase (Figure 1). The TUG and 10-meter walking test scores were positively correlated with the step width, stride time, stance phase, double-limb support phase, CV of stride time, and CV of stride length.

The K-MMSE and FAB scores were positively correlated with gait velocity, stride length, and swing phase. The K-MMSE and FAB scores were negatively correlated with the stance phase, double-limb support phase, CV of stride time, and CV of stride length.

### Model performance for predicting the risk of falling in INPH

**FIGURE 2:**
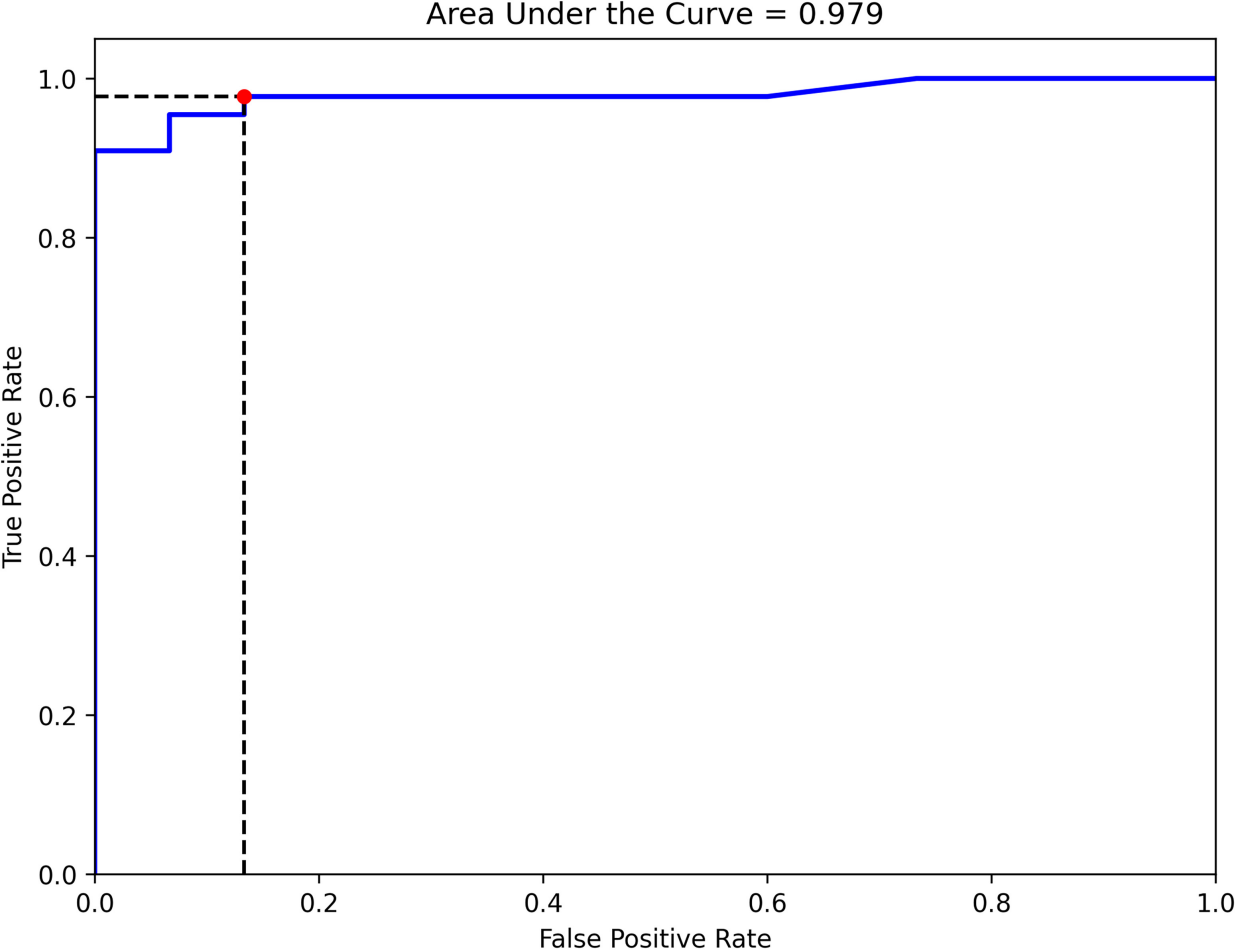
Receiver operating characteristic (ROC) curve for classifying INPH patients into high- and low-risk groups for falling using gait parameters from the vision-based gait analysis system.

The final ensemble algorithm was constructed using automated machine learning techniques provided by Mljar. We simultaneously tested 11 gait parameter predictors to discriminate between 2 groups of INPH patients classified by the TUG test as being at high risk or low risk for falling. The area under the ROC curve is the main metric for evaluating the overall classification model fit. The area under the ROC curve was 0.979, indicating that the observed fit had an excellent discriminant ability (Figure 2). We note that several subsidiary indicators, known to be sensitive to specific fit characteristics in the context of varying sample features (e.g., group sizes and balance), provide complementary information. The observed metrics are as follows: classification accuracy = 0.93, precision = 0.95, recall = 0.95, specificity = 0.87, and F1 score = 0.95.

### Model explanation for predicting the risk of falling in INPH

**FIGURE 3:**
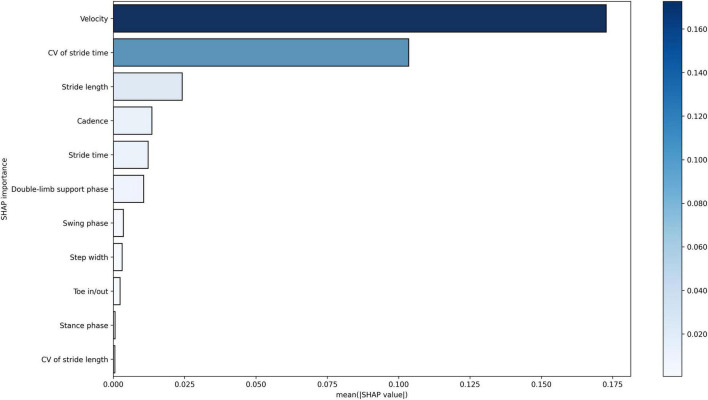
Feature importance of variables as interpreted by SHAP for classifying INPH patients into high- and low-risk groups for falling using gait parameters from the vision-based gait analysis system. CV, coefficient of variability.

**FIGURE 4:**
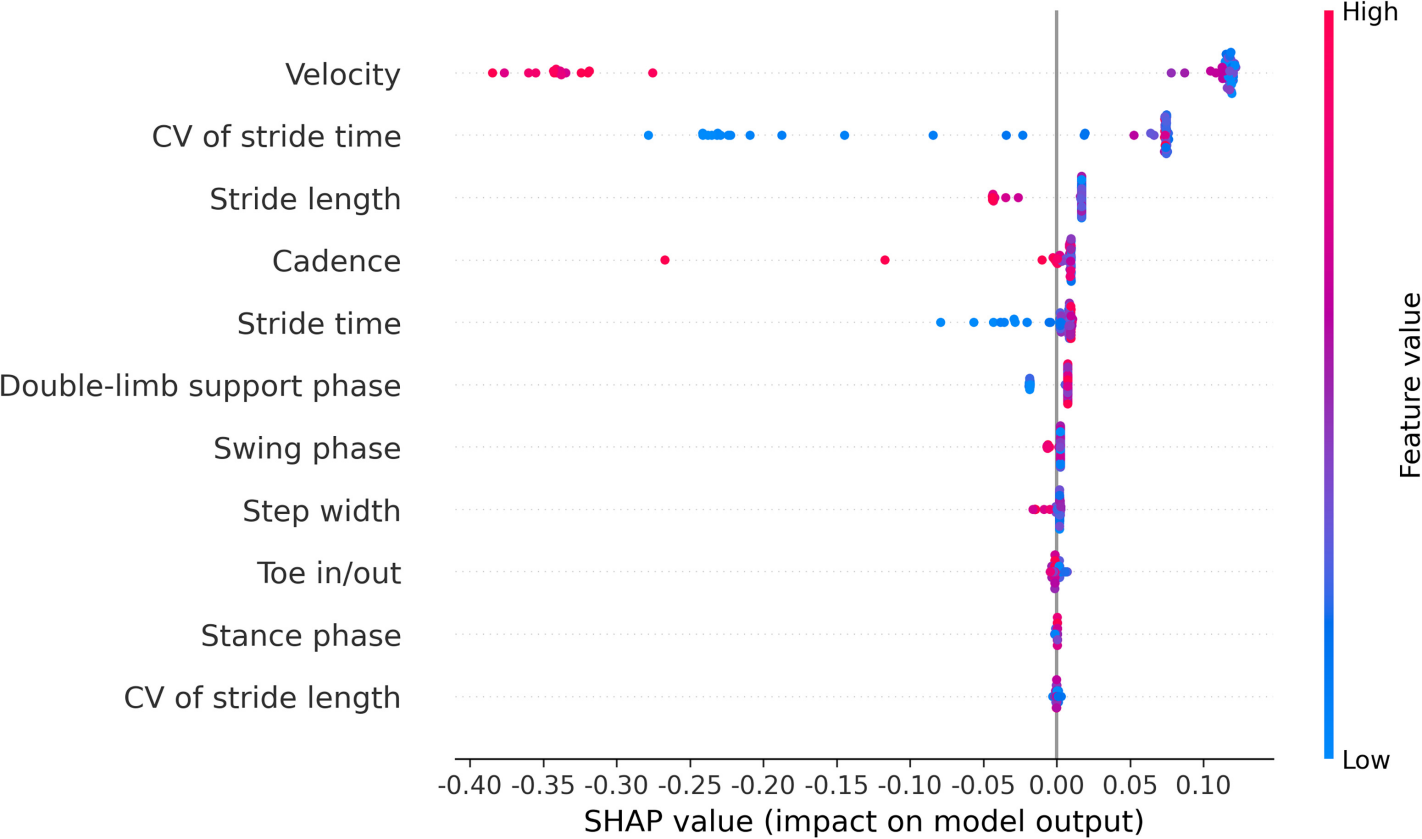
Attributes of characteristics as interpreted by SHAP for classifying INPH patients into high- and low-risk groups for falling using gait parameters from the vision-based gait analysis system. The abscissa represents the SHAP value for each feature. The red dots signify higher eigenvalues, while the blue dots indicate lower eigenvalues. CV, coefficient of variability.

The SHAP analysis yielded invaluable and pivotal insights for predicting the risk of falling in INPH. In this context, the crucial factors, ranked by importance, are gait velocity, CV of stride time, stride length, cadence, stride time, and double-limb support phase. Figures 3, 4 illustrate SHAP analysis results.

## Discussion

In this study, 10 out of 11 gait parameters measured by the vision-based gait analysis system showed correlations with clinical tests commonly used in INPH research to assess gait and balance performance (TUG and 10-meter walking test), while 7 out of 11 gait parameters were correlated with cognitive performance tests frequently employed in INPH studies (FAB and K-MMSE).

As an explanation for the association between quantitative gait analysis and clinical gait tests in our INPH patients, we might speculate as follows. The 10-meter walking test is a simple and commonly-used gait assessment in INPH, while the TUG test, which also incorporates balance control, provides a similarly straightforward yet more comprehensive evaluation (Sundstrom et al., 2022). The outcome measure for both clinical tests is the time required to complete the task, with a longer time indicating poorer performance (Sundstrom et al., 2022). Moreover, the gait of INPH patients is characterized by lower velocity, shorter stride length, a more broad-based gait, a longer stance phase with increased double-limb support, and greater gait variability compared to healthy controls (Lim et al., 2019). Our findings on the association between temporo-spatial gait parameters and clinical gait tests align with these previous results. Interestingly, gait dysfunction, as assessed by the TUG and 10-meter walking tests, was most strongly associated with worse stride time variability measured by the vision-based gait analysis system, with stride time variability showing a stronger correlation with the TUG score than with the 10-meter walking test score. A loss of consistency in the ability to produce a steady gait rhythm, resulting in higher stride-to-stride variability, has been associated with balance impairments leading to falls (Balasubramanian et al., 2009; Lim et al., 2019). It was reported that increased stride-to-stride variability in stride time was significantly correlated with a high risk for falling in community dwelling older adults (Hausdorff et al., 1997; Lim et al., 2019). Falls are also important clinical problems in patients with INPH (Lim et al., 2019). Increased gait variability has been suggested as one of the main risk factors for falls in INPH patients (Lim et al., 2019). Measurements of various temporo-spatial gait parameters offer advantages in understanding patients’ gait pathology, detecting gait disorders, identifying balance characteristics, monitoring disease progression, and evaluating medical gait interventions (Vítečková et al., 2020). Our observations suggest the potential utility of spatial and temporal parameters measured by a vision-based gait analysis system in quantifying gait impairment in INPH patients.

As an explanation for the association between quantitative gait analysis and clinical cognitive tests in our INPH patients, we might speculate as follows. Gait is a complex function regulated by the integration of multiple brain regions interconnected through white matter tracts (de Laat et al., 2011). Therefore, gait disturbances can result from lesions affecting either cortical or subcortical white matter regions (Wang et al., 2008). Further, cognitive impairment is considered a continuum from normal aging to advanced dementia, and similarly, deterioration of gait is a continuum that coexists with declines in cognition (Montero-Odasso et al., 2012). The association of cognitive and motor impairments in neurodegenerative diseases is thought to result from damage to common brain networks shared by cognitive and motor control processes (Montero-Odasso et al., 2017). Our findings were also consistent with these previous reports. Interestingly, poor cognitive performance, as assessed by the FAB and K-MMSE, was most strongly associated with reduced stride length, followed by decreased gait velocity, as measured by the vision-based gait analysis system. The pathophysiological mechanisms in INPH may involve both white matter and the cortex (Kang et al., 2020). When investigating underlying pathophysiological mechanisms involved in INPH, the cortex is usually overlooked and white matter is often the main focus of consideration (Kang et al., 2020). However, some studies suggest that when damage occurs to an axon in the brain, neuronal degeneration not only proceeds distally (Wallerian degeneration) but also proximally (dying back) (Kang et al., 2020). Cerebral hypoperfusion is also known to be related to neuronal degeneration, and is often observed in patients with INPH (Kang et al., 2020). Although the origin of gait disturbance in INPH remains unclear, the associations between specific gait parameters and performance on clinical cognitive tests suggest overlapping processes underlying these functions. Considering the fact that spatial parameters (e.g., stride length and velocity) are related to cortical areas, particularly the prefrontal cortex, while cadence and step time rely more on the brainstem and spinal cord (Valkanova et al., 2018), our findings are not surprising.

The integration of gait analysis with comprehensive neuropsychological assessment has gained increasing recognition in INPH evaluation. Recent studies have demonstrated that multimodal assessment approaches, incorporating neuroimaging markers such as callosal angle measurements, correlate significantly with tap-test response and treatment outcomes (Pyrgelis et al., 2022). Additionally, neurophysiological methods including EEG have shown that CSF drainage can improve cortical function, with changes in brain activity patterns correlating with clinical improvement (Micchia et al., 2022). These findings support our comprehensive gait assessment approach and suggest that quantitative gait parameters may serve as valuable biomarkers alongside traditional clinical measures.

A critical consideration in INPH assessment is the high prevalence of concomitant Alzheimer’s disease pathology, which may influence both cognitive and motor outcomes. Studies have shown that amyloid pathology is approximately 40% of INPH patients, with higher Tau protein levels associated with significantly worse cognitive performance (Pyrgelis et al., 2024). This underlying neurodegenerative pathology may also contribute to gait dysfunction severity and treatment responsiveness, emphasizing the importance of comprehensive assessment strategies that consider both motor and cognitive domains in patient evaluation and prognosis.

Our key finding was that an automated machine learning-based ensemble model incorporating 11 temporo-spatial gait parameters demonstrated high sensitivity and specificity in differentiating between 2 groups of INPH patients at high risk and low risk for falling, with an area under the ROC curve of 0.979, indicating excellent discriminatory ability. Further, the SHAP analysis showed a significant influence of gait velocity, CV of stride time, stride length, cadence, stride time, and double-limb support phase, in decreasing order, on the model’s classification performance. Previous studies have reported that elderly individuals at a higher risk of falling exhibit significantly lower gait velocity, greater variability in stride time, shorter stride length, lower cadence, increased stride time, and prolonged double-limb support compared to those with a lower risk of falling (Hausdorff et al., 1997; van Schooten et al., 2016; Kwon et al., 2018; Urbanek et al., 2023). Our study on INPH patients is consistent with these previous findings. Gait analysis parameters can be highly useful for assessing and quantifying gait abnormalities, as well as predicting fall risk. Moreover, a vision-based gait analysis system offers clinicians a low-cost, non-intrusive, and user-friendly tool for quantitative gait assessment in INPH patients.

Our vision-based system offers several practical advantages for clinical implementation. In outpatient clinics, the method could be seamlessly integrated into routine neurological assessments, providing objective gait measurements without requiring specialized equipment or extensive training. For pre-shunt evaluation, the system could serve as a standardized assessment tool to quantify baseline gait dysfunction and help identify surgical candidates. Post-shunt monitoring could benefit from regular gait assessments to objectively track improvement and detect potential complications or shunt malfunction over time. Beyond routine clinical monitoring, this technology presents substantial potential as a biomarker for treatment responsiveness. Our gait analysis method could provide quantitative metrics that correlate with clinical improvement following CSF tap tests. The objective nature of vision-based gait assessment may offer more sensitive detection of subtle improvements compared to subjective clinical scales, potentially enhancing patient selection for shunt surgery. Future studies should investigate the correlation between gait parameter changes post-tap test and long-term shunt outcomes. To realize this clinical potential, strategic implementation approaches will be essential. Our vision-based gait analysis system could be deployed through user-friendly platforms such as clinical kiosks or mobile applications, enabling widespread adoption across different healthcare settings. Home monitoring applications could facilitate longitudinal tracking of gait changes, supporting both clinical decision-making and research into disease progression.

We consecutively included INPH patients from a prospectively collected registry. To minimize bias in the assessment before the CSFTT, various validated objective scales were utilized. One limitation of this study is that gait variability was assessed based on a relatively small number of steps. Although we increased the number of walking trials to 4 to mitigate this, longer walking distances might be required for a more reliable evaluation. A second limitation of this study is that we did not correlate our gait parameters with actual treatment outcomes following shunt surgery, which would have provided the strongest evidence for the diagnostic and prognostic value of our method. Such correlation analysis with post-surgical outcomes in patients who underwent shunt placement would have truly demonstrated the clinical utility of vision-based gait assessment as a biomarker for treatment responsiveness and long-term prognosis. Additionally, we included INPH patients regardless of their response to the CSFTT. This approach was chosen to adhere to the international diagnostic guidelines for INPH (Relkin et al., 2005), which recommend diagnosis based on a combination of clinical history, neurological examination, and neuroimaging findings. These international INPH guidelines suggested that treatment responsiveness should not be the sole criterion for diagnosing INPH (Relkin et al., 2005). Gait assessment remains a critical component in evaluating INPH patients. Nevertheless, according to the Japanese clinical guideline (Ishikawa et al., 2008), improvement following the CSFTT provides additional diagnostic value, facilitating a transition from a “possible” to a “probable” diagnosis. Moreover, a positive response to the CSFTT is commonly used as an indicator for proceeding with shunt surgery (Ishikawa et al., 2008). Our results underscore the need for future investigations with larger and more diverse cohorts, including both responders and non-responders to the CSFTT, using quantitative gait parameters derived from vision-based gait analysis. These studies may help determine whether deep learning techniques applied to monocular video recordings can serve as objective biomarkers for predicting CSFTT responsiveness. A third limitation is an absence of quantitative neuroimaging analysis in our INPH cohort. Integrating objective gait assessments with advanced neuroimaging metrics in future studies may provide valuable insights into potential associations and underlying pathophysiological mechanisms. A fourth limitation of this study is the relatively small cohort size (*n* = 59) and the imbalance between high- and low-risk groups, which may introduce bias into our model performance estimates. To mitigate these concerns, we implemented 5-fold cross-validation to provide more robust performance estimates. The class imbalance issue was also partially addressed by the stratification strategy in cross-validation. Additionally, a base algorithm for ensembling, Random Forest, inherently employs bootstrapping techniques that enhance model robustness when working with limited sample sizes. The inclusion of these bootstrapping-based algorithms contributed to their selection in our final ensemble model. We acknowledge that larger datasets would benefit both statistical power and machine learning model performance. To address this limitation, we are planning prospective multi-center studies for external validation, which will allow us to: (1) validate our findings on independent cohorts, (2) increase sample sizes to improve statistical reliability, and (3) assess model generalizability across different clinical settings. These future validation studies will be essential for clinical translation of our proposed model. Fifth, our inclusion criteria specified only that patients be older than 40 years, resulting in a broad age range that may have introduced heterogeneity in gait characteristics unrelated to INPH pathology. This decision was made because we followed the widely used criteria of Relkin et al. (2005). In clinical practice, gait disturbance in patients with INPH is often multifactorial, and gait evaluation is commonly performed even in such cases. A more precise age stratification, however, would have enhanced the homogeneity of our study population. Sixth, although we recruited participants with gait disturbance lasting 6 months or longer according to the criteria of Relkin et al. (2005), we did not systematically analyze the exact duration of symptoms, which may be an important factor influencing the severity of gait dysfunction and treatment responsiveness. These limitations highlight the need for larger, more homogeneous cohorts with systematically collected fall events, as well as detailed clinical follow-up and treatment outcome data, to validate the clinical utility of vision-based gait analysis in INPH management. Finally, in this study, we used conventional (non-deep learning) machine learning models to predict fall risk, whereas gait parameter measurements were obtained using deep learning-based methods. The rationale for the experiment’s design is partly based on the fact that machine learning models such as Random Forest and XGBoost are known to be effective on structured tabular datasets compared to deep learning (Shwartz-Ziv and Armon, 2022), although recent deep learning models have been developed to deal with tabular datasets (Arik and Pfister, 2021). Furthermore, since deep models require many data samples to train their weights due to a large number of parameters, this approach was not suitable for our study. Despite a relatively small sample size and class imbalance between low- and high-risk groups, the experimental results demonstrated robust performance in predicting risk levels.

This study identified important associations between temporo-spatial gait parameters measured by a vision-based gait analysis system using monocular videos and TUG scores in INPH patients, with gait dysfunction, as assessed by the TUG test, showing the strongest association with increased stride time variability. An automated machine learning model based on gait parameters measured by a vision-based system can predict falling risk with excellent performance in INPH patients. According to SHAP analysis, gait velocity, stride time variability, stride length, cadence, stride time, and double-limb support phase might be important factors associated with fall risk. We suggest that our vision-based gait analysis method using monocular videos has the potential to bridge the gap between laboratory testing and clinical assessment of gait and balance in INPH patients. Our vision-based gait analysis system shows promise as an objective and practical tool for INPH management, with potential applications in routine assessments, pre- and post-shunt evaluation, and as a biomarker for treatment responsiveness. Future studies should validate these applications in larger, more homogeneous cohorts with systematically collected fall events and long-term outcome data.

## Data Availability

The raw data supporting the conclusions of this article will be made available by the authors, without undue reservation.
